# Electrical Step‐Edge Contact to a Topological Superconductor Candidate 2M‐WS_2_

**DOI:** 10.1002/advs.202508242

**Published:** 2025-11-14

**Authors:** Qikang Gan, Junwei Song, Hailing Guo, Yuqiang Fang, Xinyan Li, Han Gao, Yiwei Li, Dongdong An, Yujian Zhu, Yangchen He, Kenji Watanabe, Takashi Taniguchi, Yuzheng Guo, Qinghua Zhang, Lin Gu, Daniel A. Rhodes, Zhongkai Liu, Lede Xian, Fuqiang Huang, Lei Wang

**Affiliations:** ^1^ National Laboratory of Solid‐State Microstructures Collaborative Innovation Center of Advanced Microstructures School of Physics Nanjing University Nanjing 210093 China; ^2^ School of Electrical Engineering and Automation Wuhan University Wuhan 430072 China; ^3^ Tsientang Institute for Advanced Study Hangzhou 310024 China; ^4^ School of Materials Science and Engineering Shanghai Jiao Tong University 800 Dongchuan Road Shanghai 200240 China; ^5^ Beijing National Laboratory for Condensed Matter Physics Institute of Physics Chinese Academy of Sciences Beijing 100190 China; ^6^ School of Physical Science and Technology ShanghaiTech University Shanghai China; ^7^ Institute for Advanced Studies Wuhan University Wuhan Hubei 430072 China; ^8^ Department of Materials Science and Engineering University of Wisconsin Madison WI 53706 USA; ^9^ Research Center for Electronic and Optical Materials National Institute for Materials Science 1‐1 Namiki Tsukuba 305‐0044 Japan; ^10^ Research Center for Materials Nanoarchitectonics National Institute for Materials Science 1‐1 Namiki Tsukuba 305‐0044 Japan; ^11^ Beijing National Center for Electron Microscopy and Laboratory of Advanced Materials Department of Materials Science and Engineering Tsinghua University Beijing 100084 China; ^12^ Max Planck Institute for the Structure and Dynamics of Matter Luruper Chaussee 149 22761 Hamburg Germany; ^13^ State Key Laboratory of High Performance Ceramics and Superfine Microstructure Shanghai Institute of Ceramics Chinese Academy of Sciences Shanghai 201899/200050 China; ^14^ Jiangsu Physical Science Research Center Nanjing 210093 China

**Keywords:** 2M WS2, anisotropy, multigap, step‐edge contact, topological superconductor

## Abstract

A topological superconductor (TSC), characterized by a topologically nontrivial bulk state and protected gapless boundary states, is a promising platform for hosting Majorana bound states. However, many TSCs are environmentally sensitive, especially when thinned to 2D atomic layers. This instability poses a major challenge for integrating TSCs into electronic devices. Addressing it requires full encapsulation and high‐quality electrical contact to the TSC layer, which have not yet been achieved. Here, a novel contact geometry is demonstrated for an encapsulated topological superconductor candidate, 2M‐WS_2_, where metal electrodes contact the exposed step‐like edges with a width of only a few nanometers. This structure yields exceptionally low contact resistance (*R*_*C*_), down to ∼ 670 Ω·µm for a single unit and ∼ 65 Ω·µm for a six‐unit 2M‐WS_2_ device. Below the superconducting critical temperature (*T*_*C*_), the TSC–metal interface becomes highly transparent, as evidenced by the Andreev reflection. Furthermore, the step‐edge contact prevents contamination during fabrication, enabling unprecedentedly high‐quality devices. In encapsulated 2M‐WS_2_, twofold rotational symmetry of the critical current (*I*_*C*_) and multiple anomalous peaks are observed in the differential resistance (*dV*/*dI*). The anisotropic *I*_*C*_ originates from Fermi velocity variations along in‐plane lattice directions, while the anomalous peaks suggest multigap superconductivity in 2M‐WS_2_. These results reveal the intrinsic properties of 2M‐WS_2_ and offer a new path toward high‐performance TSC‐based electronics.

## Graphical abstract

This work develops a step‐edge contact technique for encapsulated 2M‐WS2, effectively protecting the material from contamination and achieving ultralow contact resistance ( 65 µm). Highly transparent interfaces, as evidenced by Andreev reflection, enable the observation of intrinsic superconducting properties. These include twofold symmetric critical current and multiple anomalous resistance peaks, with the latter suggesting multigap superconductivity.

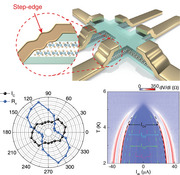

## 1 Introduction

Superconductivity and topology are two essential focuses in condensed matter physics.^[^ ^1^, ^2^, ^3^, ^4^, ^5^, ^6^ ^]^ Combining them gives rise to a novel quantum phase – topological superconductivity – which features topologically protected gapless surface states in addition to the properties of conventional superconductors. Topological superconductors (TSCs) can host Majorana zero‐energy modes, offering the potential to realize topological quantum computation due to their non‐Abelian anyonic characteristics. Currently, TSCs may possibly be achieved through two distinct approaches. One involves materials with possible intrinsic topological superconductivity, such as Cu_*x*_Bi_2_Se_3_^[^ ^1^, ^2^ ^]^ and Sn_1 − *x*_In_*x*_Te.^[^ ^7^, ^8^ ^]^ The other employs hybrid systems, consisting of a topological insulator proximitized by a conventional *s*‐wave superconductor, such as Bi_2_Te_3_^[^ ^3^, ^4^ ^]^ and HgTe.^[^ ^9^, ^10^, ^11^ ^]^ An intriguing example is 2M‐WS_2_, which features intrinsic bulk *s*‐wave superconductivity and topological surface states arising from band inversion,^[^ ^12^, ^13^, ^14^, ^15^, ^16^ ^]^ making it a promising candidate for a topological superconductor. In light of experimental evidence indicating the presence of Majorana bound states, the emergence of 2M‐WS_2_ has sparked significant research interest.^[^ ^13^ ^]^ Scanning tunneling microscopy has revealed probable Majorana bound states within magnetic vortices, and distinctive topological surface states have been observed using laser‐based angle‐resolved photoemission spectroscopy.^[^ ^13^, ^14^, ^15^ ^]^ However, the intrinsic superconducting properties of 2M‐WS_2_ have not been thoroughly investigated via electrical transport measurements due to poor device quality. In many previous studies,^[^ ^16^, ^17^, ^18^ ^]^ contact electrodes were directly deposited on the top surface of 2M‐WS_2_ flakes. In this process, the top surface of the 2M‐WS_2_ layer must be left exposed to the ambient atmosphere, lithographic resists, organic solvents, and other potential contaminants. Although BN encapsulation of 2M‐WS_2_ has been attempted,^[^ ^19^ ^]^ the contact structures still introduce impurities, affecting the measurement of its intrinsic physical properties.

For encapsulated 2D material devices, the electrical edge‐contact geometry has been extensively utilized for graphene due to its exceptional contact properties and the complete separation of the metal deposition process from van der Waals heterostructure assembly.^[^ ^20^, ^21^ ^]^ However, while similar edge‐contact approaches have been explored for other 2D materials such as transition metal dichalcogenides (TMDs),^[^ ^22^, ^23^ ^]^ the resulting contact quality has consistently fallen short of the performance achieved in graphene, often necessitating reliance on conventional top‐contact configurations. In this work, we introduce a novel contact geometry for encapsulated 2M‐WS_2_, a promising candidate for topological superconductivity. This approach extends beyond the conventional edge‐contact scheme by forming a hybrid interface where the metal electrode contacts both the material edge and a several‐nanometer‐wide region of the surface. We refer to this novel configuration as a “step‐edge contact.” This unique configuration combines the advantages of facilitated fabrication and high electrical performance, offering a new strategy for achieving reliable electrical contacts in 2D topological superconductor systems. In such high‐quality devices, we observe a twofold symmetry of the superconducting critical current corresponding to the lattice structure and multigap features in the differential resistance spectrum.

## 2 Results and Discussion

**Figure 1:**
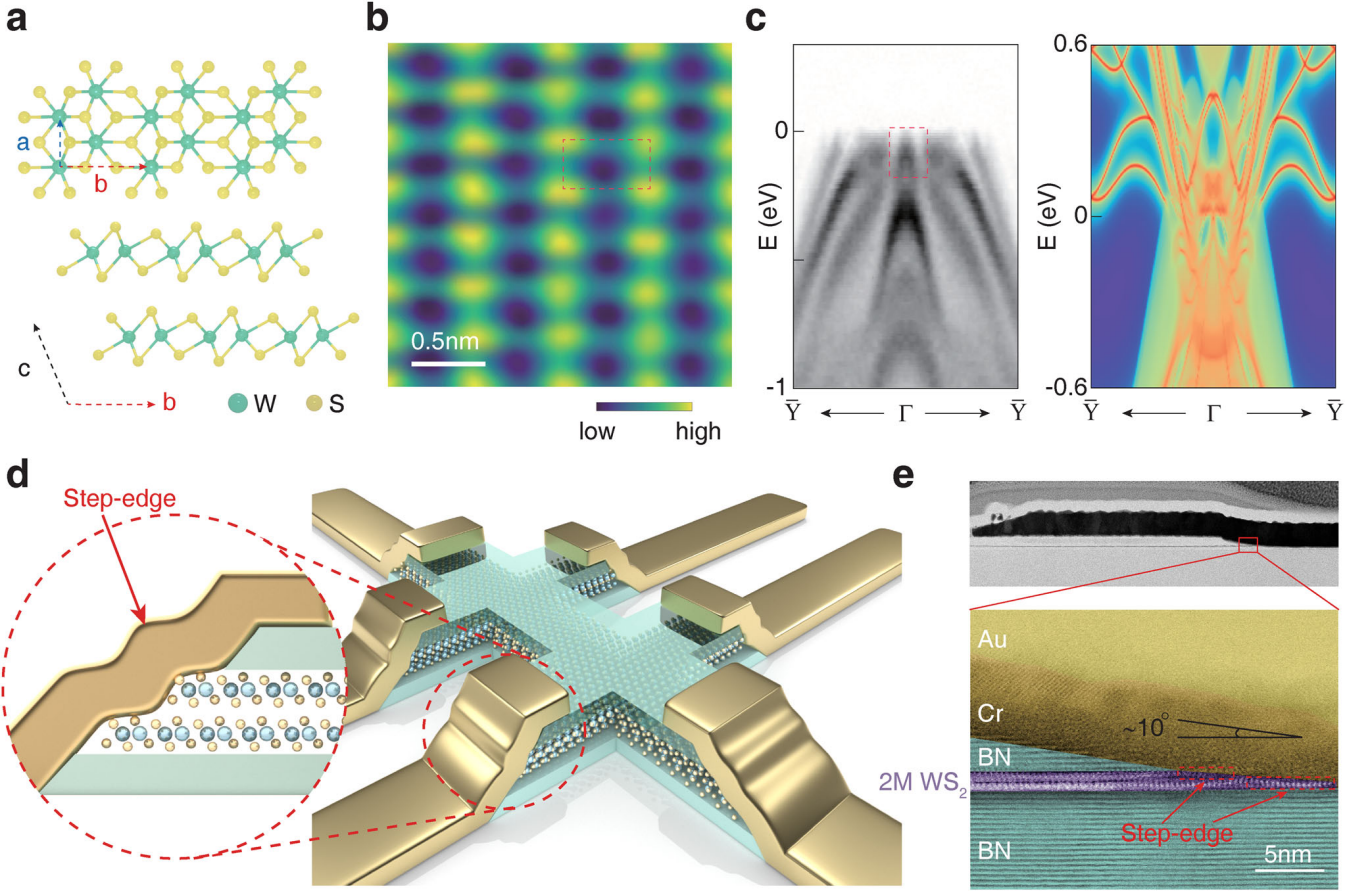
Electrical step‐edge contact geometry for 2M‐WS_2_. a) The atomic structure of 2M‐WS_2_. b) Surface atomic morphology of 2M‐WS_2_ imaged by lateral force microscopy, 2.5 nm × 2.5 nm. c) High‐resolution ARPES band dispersion along the $\bar{Y}-\Gamma -\bar{Y}$ direction (left) of bulk 2M‐WS_2_ and the calculated energy band (right) of two units 2M‐WS_2_. d) Cartoon of the electrical step‐edge contact geometry. e) High‐resolution STEM images showing details of the electrical step‐edge contact geometry.

Figure **1**a shows the 2M‐WS_2_ lattice structure, consisting of layers stacked in a distorted octahedral layout and shares an identical monolayer crystal structure with 1T′ WTe_2_. Unlike the 1T′ structure, in which adjacent layers undergo a glide mirror operation, the neighboring layer in 2M stack translates along the *b* direction. Consequently, inversion symmetry preserves in atomically thin 2M‐WS_2_, exhibiting a centrosymmetric structure with a space group of *C*_2/*m*_ in monoclinic symmetry. Using lateral force microscopy (LFM), we illustrate the surface atomic morphology of thin 2M‐WS_2_ flake in Figure 1b. The highlighted area displays a rectangular structure (red dashed line), corresponding to a top view of the primitive cell, with dimensions of the long and short axes consistent with previous studies.^[^ ^12^, ^13^, ^16^ ^]^ We further confirm the 2M phase by taking its Raman spectrum, which exhibits a series of peaks at 112, 178, 270, 316, and 407 cm^−1^ (Figure S1, Supporting Information).^[^ ^12^ ^]^

As depicted in Figure 1c, we perform synchrotron‐based ARPES measurements of the 2M‐WS_2_ band structure. The red dashed rectangle illustrates the band inversion between the p orbital of sulfur (S) atoms and the d orbital of tungsten (W) atoms, resulting in a topological nontrivial gap in the bulk. Previous theoretical studies have predicted that single unit 2M‐WS_2_ features nontrivial edge states.^[^ ^24^ ^]^ Here, our calculations (using the WannierTools code^[^ ^25^ ^]^) further demonstrate 2M‐WS_2_ is topological nontrivial in its two and three units structures, as illustrated in right panel of Figure 1c and Figure S2 (Supporting Information). Along the Y‐edge, the edge states are distinctly separated in momentum space from the bulk states, a characteristic feature of topologically protected boundary modes. Notably, the edge states exhibit dispersion that crosses the Fermi level, further distinguishing them from the bulk states. Our device structure is shown in Figure 1d, where the 2M‐WS_2_ is encapsulated between two BN layers. This heterostructure is patterned to a Hall‐bar shape by plasma etching, with the exposed step‐shaped edge (red arrows) of the 2M‐WS_2_ electrically contacted by metallization layers directly. In Figure 1e, we present High‐resolution scanning transmission electron microscope (STEM) images of the cross‐section of contact structure (upper panel). The lower panel shows the false‐color STEM image zoomed in at edge region, which reveals that BN/2M‐WS_2_/BN interfaces are atomically clean and Cr layer covers the step‐shaped edge (red dashed rectangles) of the 2M‐WS_2_. This structure is achieved by enhanced etching selectivity between BN and 2M‐WS_2_, the resulting etching angle is as small as ∼ 10 degrees (blue arrow) which is much more acuter than the etching angle of graphene edge contact, 50 degrees.^[^ ^20^ ^]^ The edge nature of the metal contact is confirmed within the resolution of the STEM image, as there is no evidence of metal atom diffusion at the 2M‐WS_2_/BN interfaces.

**Figure 2:**
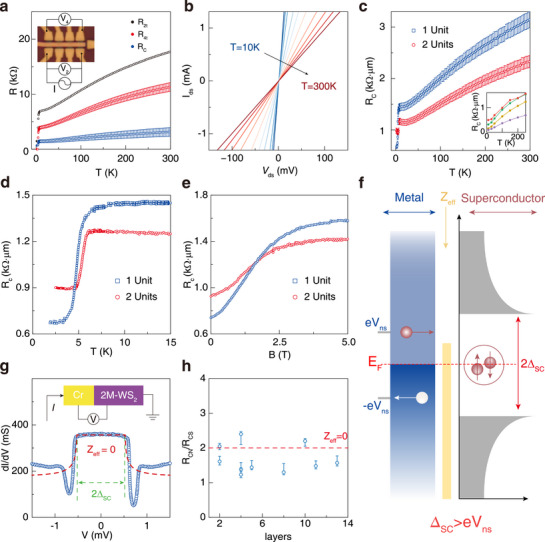
Contact resistance measurements. a) Two terminal resistance (*R*_2*t*_), four terminal resistance (*R*_4*t*_) and extracted contact resistance (*R*_*C*_) as a function of temperature (device A, 1 unit). Inset shows an optical image of a Hall‐bar shape device with electrical edge‐contacts. b) Two terminal *I–V* measurement of the device (device B, 5 unit) at various temperatures. The linear dependence indicates a typical ohmic contact at all temperature range. c) Comparison of contact resistance between 1 unit (device A) and 2 units (device C) devices. Contact resistances of thicker devices are shown in inset, red (green, yellow, purple) curve represents 3 (4, 5, 6) units. The contact resistance is as low as 65 Ω·µm for six units device. d,e) The *R*_*C*_ of 1 (device A) and 2 (device C) units devices as a function of temperature ranging from 2 to 15 K (d) and out‐of‐plane magnetic field ranging from 0 to 5 T (e). f) Illustration of Andreev reflection process at finite effective barrier strength. The AR process must satisfy Δ_*SC*_ > *eV*_*ns*_. g) Differential conductance versus bias voltage for a Cr‐2M‐WS_2_ interface (blue circle, device D, 5 units). The dashed red line is the BTK theory fit with *Z*_*eff*_ = 0. Inset: schematic illustration of the measurement setup. h) The *Q*(*R*_*CN*_/*R*_*CS*_) versus thickness of 2M‐WS_2_ for all devices. The red dashed line indicates the zero effective barrier strength.

We next characterize the contact resistan ce *R*_*C*_ of this electrical step‐edge contact geometry. **Figure** **2**a inset shows an optical image of our hall‐bar shape device, on which we can perform two‐ and four‐terminal resistance (*R*_2*t*_, *R*_4*t*_) measurements (other device images are shown in Figure S3, Supporting Information). *R*_4*t*_ directly reflects the channel resistance while *R*_2*t*_ consists of both channel resistance and two contact resistances. We use the differences between *R*_2*t*_ and *R*_4*t*_ to extract *R*_*C*_ (see Experimental Section for details). Figure 2a plots *R*_2*t*_, *R*_4*t*_ and *R*_*C*_ as a function of temperature for a single unit (2 layers) 2M‐WS_2_ (device A). From room temperature (*T* = 300 K) to the superconducting transition point (*T*_*C*_ = 6 K), *R*_*C*_ decreases approximately linearly from 3 kΩ·µm to 1.45 kΩ·µm. Notably, the *I–V* curves (Figure 2b) in this temperature range exhibit linear behavior, indicating the formation of ohmic contact between metal electrode and 2M‐WS_2_. As temperature further decreases, a significant step‐drop in *R*_*C*_ is observed at *T*_*C*_ (Figure S4, Supporting Information), suggesting an enhanced contact performance of the step‐edge shape superconductor/metal interface compared to the metal/metal interface. In Figure 2c, we extract *R*_*C*_ for multiple devices with different thickness (up to six units), whose temperature dependence shows same trend as single unit behavior. As the thickness increases, *R*_*C*_ decreases due to lager contact area.

We move to understand the physical mechanism why *R*_*C*_ changes drastically at the superconducting transition temperature. Figure 2d shows magnified *R*_*C*_−*T* curves around *T*_*C*_ in Figure 2a. The *R*_*C*_ of the single unit (bilayer) device decreases approximately from 1.45 kΩ·µm (normal state) to 670 Ω·µm (superconducting state), and two units (quadrilayer) device shows the similar behavior. We can also use a magnetic field to drive the device from superconducting state to normal state and observe identical changes in *R*_*C*_ values (Figure 2e), which indicates that the *R*_*C*_ step‐drop arises from the material's inherent metal‐to‐superconductor transition. In Figure 2f, we use the Andreev Reflection (AR) model to elucidate this phenomenon. When an electron enters the superconductor from a normal metal, it has to participate as a Cooper pair member. A hole with opposite spin and velocity, but equal momentum to the incident electron, reflects from the metal‐supercondutor interface. Thereby, theoretically the differential conductance at this interface enhances by two times comparing to metal‐normal state interface. In this process, the energy of incident electron (*eV*_*ns*_) needs to be less than the superconducting gap of 2M‐WS_2_ (Δ_*SC*_). In our experiment, the entire circuit operates under a constant low frequency a.c. current of 100 nA, corresponding to an energy scale corresponding to an energy on the order of ∼ 0.1 meV, which is significantly lower than the superconducting energy gap, therefor we indeed observe this “step‐drop.”

We further verify this AR model by varying the energy of incident electrons. In Figure 2g, we plot the differential conductance (*dI*/*dV*) versus the voltage drop across the contact interface. The voltage‐dependent *dI*/*dV* exhibits a typical behavior which is consistent with Andreev reflection model, as reported before.^[^ ^26^, ^27^, ^28^, ^29^ ^]^ The dashed red line corresponds to the Blonder‐Tinkham–Klapwijk (BTK) theory fit for a barrier parameter of *Z*_*eff*_ = 0. Due to the presence of additional gold wire resistance, the ratio of the sub‐gap differential conductance to the normal‐state conductance deviates slightly from the theoretical value of two. Additionally, the dip in differential conductance near the superconducting gap edge can be well captured by the multigap extension of the BTK theory,^[^ ^28^ ^]^ which lends further support to our conclusion that 2M‐WS_2_ exhibits multigap superconductivity. Nevertheless, the observation of a perfect plateau in the *dI/dV* curve within the superconducting gap region–a characteristic signature of the ideal BTK scenario–provides compelling evidence for the formation of nearly transparent contacts. To quantify this step‐drop transition process in *R*_*C*_, we define the ratio of *R*_*C*_ above and below *T*_*C*_ as *Q* (*R*_*CN*_/*R*_*CS*_). According to BTK theory, the effective barrier *Z*_*eff*_ in the metal‐superconductor interface can be estimated by *Q* ≃ 2(1 + *Z*_*eff*_)/(1 + 2*Z*_*eff*_)^2^.^[^ ^26^, ^28^ ^]^ Figure 2h displays the *Q* values for devices with different number of layers. The distribution of *Q* is around two for all thickness values with small variation. The extracted effective barrier *Z*_*eff*_ values are between 0 and 0.2 (see Figure S5, Supporting Information), further confirming a nearly transparent contact interface.^[^ ^26^ ^]^

**Figure 3:**
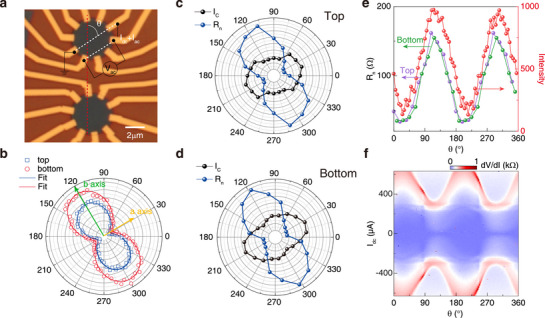
Anisotropy of 2M‐WS_2_ superconductivity. a) Optical micrograph of two encapsulated 2‐units 2M‐WS_2_ devices (device E,F) etched to a disk shape with step‐edge contact electrodes. *θ* is the angle between the measurement direction (white dashed line) and the *b*−axis of the crystal (red dashed line).These two devices are derived from the same stack. The scale bar is 2 µm. b) Angular dependence of the *A*_*g*_ phonon mode at 406 cm^−1^. Square (circle) symbols represent top (bottom) device. *A*_*g*_ intensity of both two devices along the *b*−axis (*a*−axis) reach the maximum (minimum). c,d), Angular dependence of critical current (*I*_*C*_, black circle) and normal resistance (*R*_*n*_) of two devices c) for top, d) for bottom) at 1.5 K. Normal resistance is defined as the differential resistance measured when the DC current exceeds the critical current. e) Comparison of angular dependence between *A*_*g*_ intensity and normal resistance of top (blue data) and bottom (black data) devices. f) Map of differential resistance (*dV*/*dI*) versus direct current *I*_*dc*_ and angle of parallel magnetic field (*B*_∥_) at *B*_∥_ = 10 T of top device. The direction of *I*_*dc*_ is fixed.

With such exceptional contact characteristics and unprecedented atomically clean device interfaces, we finally look into the inherent superconducting behaviors of 2M‐WS_2_. **Figure** **3**a shows a BN‐encapsulated 2M‐WS_2_ stack etched to two multi‐terminal circler disks (top and bottom) to investigate the in‐plane anisotropy. We first identify the crystal orientation by performing angle‐dependent Raman spectroscopy. Figure 3b shows the angular dependence of Raman intensities for both devices, measured at the *A*_*g*_ phonon mode around 406 cm^−1^ with polarized configuration. By comparing with the LFM results, we confirm that the Raman intensity is maximized along the *b*‐axis and minimized along the *a*‐axis, with additional details in Figure S6.

Such anisotropy in 2M‐WS_2_ has been predicted to modulate the superconducting properties correspondingly. However, experimental efforts aiming to measure along different crystal directions have so far been hindered by the limitations in device fabrication. With our step‐edge contact to fully encapsulated device geometry, we for the first time measure the differential resistance (*dR* = *dV*/*dI*) as a function of d.c. current in multiple directions (see the Experimental section for details), from which we derive the angular dependence of both the in‐plane critical current, *I*_*C*_ (defined as the d.c. current at which *dV*/*dI* reaches zero) and the normal resistance, *R*_*n*_ (defined as the differential resistance when the d.c. current exceeds the critical current). The angle *θ* represents the angle between the measurement direction (white dashed line) and the vertical axis (red dashed line). The black (blue) data points in Figure 3c depict the angular dependence of *I*_*C*_ (normal resistance *R*_*n*_) for the top device, both of which display a clear twofold rotational symmetry. A 90‐degree rotation is observed between *I*_*C*_ and *R*_*n*_, where the maximum of *R*_*n*_ and the minimum of *I*_*C*_ both align along approximately at 120°. Notably, the symmetry of the patterns remains consistent across both the top and bottom devices (Figure 3d), confirming the reliability of our data and the homogeneity of the device. While previous ARPES studies have reported an isotropic superconducting gap,^[^ ^14^ ^]^ confirmed with our isotropic critical temperature (*T*_*c*_) measurement results (see Figure S7, Supporting Information), the observed anisotropy in *I*_*C*_ is likely a result of variations in the Fermi velocity along the two primary in‐plane lattice directions. Figure 3e presents comparative plots of the Raman peak intensity and normal resistance as a function of angle for the top and bottom devices. The nearly identical trends in variation indicate that the anisotropy in *I*_*C*_ and *R*_*n*_ is indeed linked to the underlying crystal symmetry. In addition, we study the anisotropy characteristic of 2M‐WS_2_ under various *B*_∥_ directions in Figure 3f while the direction of *I*_*dc*_ is fixed. Under a fixed direct current, the *dR* exhibits periodic oscillations as a function of the in‐plane magnetic field orientation angle. The two‐fold oscillation behavior observed in the angular‐dependent *dR* is consistent with the behavior of *I*_*C*_ as a function of current direction. The observed consistency between the anisotropic responses of 2M‐WS_2_ to the applied electric field and in‐plane magnetic field serves as evidence for the uniformity of both the sample and the step‐edge contact structure.

**Figure 4:**
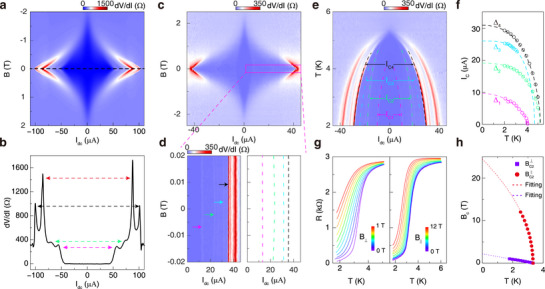
Multigap like features and enhanced critical field of 2M‐WS_2_ superconductivity. a) Map of *dV*/*dI* versus *I*_*dc*_ and B at T = 1.5 K (device G, 2 units). It is noticed that *I*_*C*_ values for positive and negative directions are not exactly symmetric, implying possible superconducting diode effect. b) Differential resistance spectra of device G at zero magnetic field (black dash line in a). c) Map of *dV*/*dI* versus *I*_*dc*_ and B at T = 1.5 K (device H, 3 layers). d) Left: The magnified region in c) with arrows pointing four peaks. Right: Boundaries of four diamond‐shaped regions in d) left panel. e) Map of *dV*/*dI* versus *I*_*dc*_ and temperature. Dashed lines delineate the temperature dependence of four distinct peaks. f) Temperature dependence of all four critical currents *I*_*c*_. The dependence can be fitted by *I*_*c*_(*t*)/*I*_*c*_(0) = (1 − *t* ^2^)^α^(1 + *t* ^2^)^β^, where *t* = *T*/*T*_*C*_. The fitting curves are plotted as the dashed lines. g) Temperature dependence of the resistance measured under various out‐of‐plane (*B*_⊥_, left) and in‐plane (*B*_∥_, right) magnetic field (device G, 1 unit). h) Extracted critical magnetic field *B*_*C*_ as a function of critical temperature *T*_*C*_ from g). Square symbols and circle symbols represent *B*_⊥_ and *B*_∥_, respectively. The violet dashed line is the linear fit to $B_{C}^{⊥}=\phi_{0}/\left(2\pi \xi {\left(0\right)}^{2}\right)\left(1-T/T_{C}\right)$. The red dashed line is the theoretical fit to $B_{C2}^{∥}=\left(\phi_{0}\sqrt{12}\right)/\left(2\pi \xi \left(0\right)d\right)\sqrt{(1-T/T_{c}})$.

In **Figure** **4**a, we map the *dR* as functions of d.c current and vertical magnetic field (*B*_⊥_). *I*_*C*_ and critical field (*B*_*C*_) enclose a diamond shape superconducting region without disorder/junction induced Fraunhofer oscillation patterns on the boundary. Interestingly, on such high quality device, we for the first time observe anomalous multiple peaks in the differential resistance spectra (Figure 4b), which have been interpreted as arising from multigap superconductivity in WTe_2_.^[^ ^30^ ^]^ A similar behavior has also been observed in device H, shown in Figure 4c. For clarity, we magnify the rectangle area of Figure 4c and mark the boundaries of this diamond‐shaped regions in Figure 4d left panel. There are four peaks (pointed by arrows) in *dR* at *I*_*dc*_ ranging from 8 to 35 µA (Figure 4d right panel). The *I* − *V* characteristics can be obtained by integrating *dR* − *I* curves (Figure S8, Supporting Information), through which we can estimate the corresponding energy gaps of these superconducting pocket. The gap value of the outermost peak is around 1.5 meV which is similar to the reported superconducting gap before.^[^ ^14^, ^16^ ^]^ Figure 4e shows the map of *dR* versus d.c current and temperature. The *I*_*dc*_ positions of all four peaks shift toward zero as temperature increases and exhibit the typical temperature dependence of superconducting critical current, which indicates that these peaks origin from the intrinsic superconductivity of 2M‐WS_2_. We further extract the *I*_*C*_ values (circles) of all four peaks as a function of temperature in Figure 4f. These data points can be well fitted by *I*_*C*_(*t*)/*I*_*C*_(0) = (1 − *t* ^2^)^α^(1 + *t* ^2^)^β^, which has been widely used for type‐II superconductors,^[^ ^31^, ^32^, ^33^, ^34^ ^]^ where *t* = *T*/*T*_*C*_ is the normalized temperature. The distinct critical temperatures *T*_C_ obtained from the fitting parameters of *I*_C1_ and *I*_C3_, however, deviate significantly from those of *I*_C2_ and *I*_C4_ (Table S1, Supporting Information). This discrepancy strongly suggests that the critical currents *I*_C1_ and *I*_C3_ originate from a different superconducting gap compared to *I*_C2_/*I*_C4_. Such multi‐*T*_C_ behavior is a hallmark of multigap superconductivity, where multiple superconducting condensates with distinct energy gaps coexist in the same material.^[^ ^35^ ^]^ Although alternative explanations–such as inhomogeneity, phase‐slip phenomena, or interface effects–could also give rise to multiple peaks in the differential resistance, we can rule out inhomogeneity and interface effects in our device based on the consistency observed across multiple measurement channels (Figure S9, Supporting Information). Furthermore, the length of our device exceeds the prerequisite scale for phase‐slip phenomena, thereby precluding their occurrence. Our results provide compelling evidence that 2M‐WS_2_ hosts multiple superconducting gaps, likely arising from either (i) multiple superconducting pockets or (ii) the coexistence of superconductivity in different orbital channels.^[^ ^36^ ^]^

Furthermore, we performed the magnetic responses with *B*_⊥_ up to 1 T and *B*_∥_ up to 12 T on a one unit thick sample (Figure 4g). We summarize the relationship between critical magnetic field (*B*_*C*_) and temperature in Figure 4h, where the *B*_*C*_ is defined as the magnetic field corresponding to 50% of *R*_*n*_ at every fixed temperature *T*. For out‐of‐plane critical magnet field, $B_{C2}^{⊥}$ displays a linear dependence on temperature, consistent with the linearized Ginzburg–Landau (GL) expression $B_{C}^{⊥}=\phi_{0}/\left(2\pi \xi {\left(0\right)}^{2}\right)\left(1-T/T_{C}\right)$, where *ϕ*_0_ is the magnetic flux quantum and *ξ*(0) is the zero‐temperature GL in‐plane coherence length. The fitting (purple dashed line) gives *ξ*(0) = 12.3 nm. For the in‐plane critical magnetic field, $B_{C2}^{∥}$ follows the 2D GL equation $B_{C2}^{∥}=\left(\phi_{0}\sqrt{12}\right)/\left(2\pi \xi \left(0\right)d\right)\sqrt{(1-T/T_{c}})$, where *d* is the superconducting thickness. We extract a superconducting thickness of *d* = 3.7 nm from this fitting, which however has been previously found to overestimate the actual material thickness in 2D systems.^[^ ^37^, ^38^ ^]^ Also, the extrapolated zero‐temperature $B_{C2}^{∥}$ reaches approximately 25 T, which is far beyond the Pauli paramagnetic limit for Bardeen–Cooper–Schrieffer (BCS) superconductors of this device (*B*_*P*_ = 1.86*T*_*C*_ = 6.14 T), indicating the unconventional superconducting pairing mechanism.

## 3 Conclusion

In conclusion, we demonstrate an electrical step‐edge contact geometry to an encapsulated topological superconductor candidate 2M‐WS_2_ with low contact resistance, which also enables the fabrication of high‐quality devices with atomically clean interfaces. Below *T*_*C*_, contact resistance is further reduced by nearly half thanks to Andreev reflection. With such unprecedented high quality devices, we uncover the the anisotropic response of *I*_*C*_ and *R*_*n*_ to applied electrical field and multiple superconducting pockets features in 2M‐WS_2_ for the first time. The advancements in this work provide new avenues for high quality electrical devices using 2D topological superconductors. Building upon this encapsulated architecture, we may conduct interference‐based experiments to provide evidence of gapped edge states in future investigations.

## 4 Experimental Section

### Sample Fabrication

The multi‐layer heterostructures are fabricated using “pick‐up” method^[^ ^20^ ^]^ where the 2M‐WS_2_ is encapsulated by two flakes of hexagonal boron nitride (hBN). The polyproylene carbonate (PPC) residue during this process is released by the actone. Electron‐beam lithography is applied multiple times to make an etch mask to define the Hall‐bar geometry and open the electrode slots. The etch is performed in a CHF_3_/CF_4_/O_2_ (3:1:1) plasma at 60 W to remove the redundant regions of the heterostructure and the 2M‐WS_2_ are step‐edge contacted by e‐beam evaporated thin metal layers consisting of Cr/Pd/Au (1 nm/15 nm/60 nm). Besides Cr, we also test the first metal layer touching the 2M‐WS_2_ using Pt, Pd, and Ti, which all exhibit good contact resistance too.

### Transport Measurement

The transport measurements is performed in a cyrostat cooled down to 1.5 K with a superconducting magnet. All data is documented with lock‐in amplifiers with a sourcing low‐frequency AC current *I*_*ac*_ of 100 nA at 17.777 Hz and a DC current *I*_*dc*_ . The configuration of our device comprises a Hall‐bar with contact width W = 1 µm. The resistance measured via the two‐terminal method (*R*_2*t*_) consist three components: the intrinsic resistance of the metal leads, the interface contact resistance (*R*_*C*_) and the resistance of the 2M‐WS_2_ channel (*R*_*L*_). In which *R*_*L*_ can be extracted as r*R*_4*t*_, where *R*_4*t*_ is resistance of 2M‐WS_2_ measured by four‐terminal method and r is the ratio of channel length measured in the two‐terminal and four‐terminal methods. The *R*_*C*_ is calculated as (*R*_2*t*_ − *rR*_4*t*_)/2, the metal leads resistance is ignored.

### ARPES Measurement

The ARPES measurements were performed at beamline BL07U of Shanghai Synchrotron Radiation Facility (SSRF), China, with a photon energy of 94 eV. The samples were cleaved in situ under high vacuum below 5 × 10^−11^ Torr. The data were collected by a ScientaOmicron DA30‐L analyzer. The angle and energy resolutions were below 0.2° and 30 meV, respectively.

### STEM Sample Preparation and Imaging

The cross‐sectional samples were prepared using a FEI Helios 660i focused ion beam (FIB) with a gallium ion beam source. The operating voltage of FIB was reduced from 30 to 2 kV during the FIB milling process. After sample preparation, bright‐field (BF) and high‐angle annular dark‐field (HAADF) scanning transmission electron microscopy (STEM) imaging was performed using a double Cs‐corrected JEOL JEM‐ARM200CF operated at 200 kV with the HAADF collection semi‐angles of 90–370 mrad.

### Raman Measurement

Raman spectra were performed under 532 nm laser in the Witec confocal Raman Microscope.

### LFM Measurement

LFM image were performed under lateral force microscope mode in the Oxford Cypher atom force microscope.

### Blonder–Tinkham–Klapwijk(BTK) Model

In the BTK formalism,^[^ ^26^ ^]^ the total current across the metal/2M‐WS_2_ interface is proportional to 1 + *A*(E)‐*B*(E), here, *A*(E) is the the Andreev reflection probability and *B*(E) is the reflected electron probability. Thus, the formula of differential conductance can be written as

(1) $$\frac{dI}{dV}=2N\left(0\right)e\nu_{f}S\int_{-\infty }^{+\infty }\left[\frac{df_{0}\left(E-eV\right)}{dV}\right]\left[1+A\left(E\right)-B\left(E\right)\right]dE,$$

where *N*(0) is the density of states of normal metal at the Fermi level, e is electron charge, *ν*_*F*_ is Fermi velocity of the chosen metal, *S* is the effective contact area, and *f*_0_(x) is Fermi–Dirac distribution function. Here,

(2) $$A\left(E\right)=\left\{\begin{array}{cc} \frac{\Delta^{2}}{\left(E^{2}+\left(\Delta^{2}-E^{2}\right){\left(1+2Z_{eff}^{2}\right)}^{2}\right)} & ifE<\Delta ;\\ \frac{\mu_{0}^{2}\nu_{0}^{2}}{\gamma^{2}} & ifE>\Delta ; \end{array}\right.$$

(3) $$B\left(E\right)=\left\{\begin{array}{cc} 1-A(E) & ifE<\Delta ;\\ \left(\mu_{0}^{2}-\nu_{0}^{2}\right)Z_{eff}^{2}\left(1+Z_{eff}^{2}\right)/\gamma^{2} & ifE>\Delta ; \end{array}\right.$$

(4) $$\begin{array}{c} \mu_{0}^{2}=1-\nu_{0}^{2}=(1+\left(\sqrt{E^{2}-\Delta^{2})/E^{2}}\right)/2, \end{array}$$

(5) $$\begin{array}{c} \gamma =\mu_{0}^{2}+\left(\mu_{0}^{2}-\nu_{0}^{2}\right)Z_{eff}^{2}. \end{array}$$

With this analytic expression, we can derive the expression of *Q*, in which the differential conductivity and normal conductivity are approximately equal.

## Conflict of Interest

The authors declare no conflict of interest.

## Supporting information

Supporting Information

## Data Availability

The data that support the findings of this study are available from the corresponding author upon reasonable request.

## References

[1] Y. S. Hor , A. J. Williams , J. G. Checkelsky , P. Roushan , J. Seo , Q. Xu , H. W. Zandbergen , A. Yazdani , N. P. Ong , R. J. Cava , Phys. Rev. Lett. 2010, 104, 057001. 20366785 10.1103/PhysRevLett.104.057001

[2] S. Sasaki , M. Kriener , K. Segawa , K. Yada , Y. Tanaka , M. Sato , Y. Ando , Phys. Rev. Lett. 2011, 107, 217001. 22181913 10.1103/PhysRevLett.107.217001

[3] J.‐P. Xu , C. Liu , M.‐X. Wang , J. Ge , Z.‐L. Liu , X. Yang , Y. Chen , Y. Liu , Z.‐A. Xu , C.‐L. Gao , D. Qian , F.‐C. Zhang , J.‐F. Jia , Phys. Rev. Lett. 2014, 112, 217001.

[4] J.‐P. Xu , M.‐X. Wang , Z. L. Liu , J.‐F. Ge , X. Yang , C. Liu , Z. A. Xu , D. Guan , C. L. Gao , D. Qian , Y. Liu , Q.‐H. Wang , F.‐C. Zhang , Q.‐K. Xue , J.‐F. Jia , Phys. Rev. Lett. 2015, 114, 017001. 25615497 10.1103/PhysRevLett.114.017001

[5] H. Zuo , J.‐K. Bao , Y. Liu , J. Wang , Z. Jin , Z. Xia , L. Li , Z. Xu , J. Kang , Z. Zhu , G.‐H. Cao , Phys. Rev. B 2017, 95, 014502.

[6] L. A. Wray , S.‐Y. Xu , Y. Xia , Y. S. Hor , D. Qian , A. V. Fedorov , H. Lin , A. Bansil , R. J. Cava , M. Z. Hasan , Nat. Phys. 2010, 6, 855.

[7] A. S. Erickson , J.‐H. Chu , M. F. Toney , T. H. Geballe , I. R. Fisher , Phys. Rev. B 2009, 79, 024520.

[8] S. Sasaki , Z. Ren , A. A. Taskin , K. Segawa , L. Fu , Y. Ando , Phys. Rev. Lett. 2012, 109, 217004. 23215610 10.1103/PhysRevLett.109.217004

[9] J. B. Oostinga , L. Maier , P. Schüffelgen , D. Knott , C. Ames , C. Brüne , G. Tkachov , H. Buhmann , L. W. Molenkamp , Phys. Rev. X 2013, 3, 021007.

[10] S. Hart , H. Ren , T. Wagner , P. Leubner , M. Mühlbauer , C. Brüne , H. Buhmann , L. W. Molenkamp , A. Yacoby , Nat. Phys. 2014, 10, 638.

[11] J. Wiedenmann , E. Bocquillon , R. S. Deacon , S. Hartinger , O. Herrmann , T. M. Klapwijk , L. Maier , C. Ames , C. Brüne , C. Gould , A. Oiwa , K. Ishibashi , S. Tarucha , H. Buhmann , L. W. Molenkamp , Nat. Commun. 2016, 7, 10303. 26792013 10.1038/ncomms10303 PMC4735757

[12] Y. Fang , J. Pan , D. Zhang , D. Wang , H. T. Hirose , T. Terashima , S. Uji , Y. Yuan , W. Li , Z. Tian , J. Xue , Y. Ma , W. Zhao , Q. Xue , G. Mu , H. Zhang , F. Huang , Adv. Mater. 2019, 31, 1901942. 10.1002/adma.201901942 31157482

[13] Y. Yuan , J. Pan , X. Wang , Y. Fang , C. Song , L. Wang , K. He , X. Ma , H. Zhang , F. Huang , W. Li , Q.‐K. Xue , Nat. Phys. 2019, 15, 1046.

[14] Y. Li , H. Zheng , Y. Fang , D. Zhang , Y. Chen , C. Chen , A. Liang , W. Shi , D. Pei , L. Xu , S. Liu , J. Pan , D. H. Lu , M. Hashimoto , A. Barinov , S. W. Jung , C. Cacho , M. X. Wang , Y. He , L. Fu , H. J. Zhang , F. Q. Huang , L. X. Yang , Z. K. Liu , Y. L. Chen , Nat. Commun. 2021, 12, 2874. 34001892 10.1038/s41467-021-23076-1 PMC8129086

[15] S. Cho , S. Huh , Y. Fang , C. Hua , H. Bai , Z. Jiang , Z. Liu , J. Liu , Z. Chen , Y. Fukushima , A. Harasawa , K. Kawaguchi , S. Shin , T. Kondo , Y. Lu , G. Mu , F. Huang , D. Shen , Nano Lett. 2022, 22, 8827. 36367457 10.1021/acs.nanolett.2c02372

[16] E. Zhang , Y.‐M. Xie , Y. Fang , J. Zhang , X. Xu , Y.‐C. Zou , P. Leng , X.‐J. Gao , Y. Zhang , L. Ai , et al., Nat. Phys. 2023, 19, 106.

[17] X. Che , Y. Deng , Y. Fang , J. Pan , Y. Yu , F. Huang , Adv. Electron. Mater. 2019, 5, 1900462.

[18] Y. Ji , Y. Chu , A. Zhi , J. Tian , J. Tang , L. Liu , F. Wu , Y. Yuan , R. Yang , X. Tian , D. Shi , X. Bai , W. Yang , G. Zhang , Phys. Rev. B 2022, 105, L161402.

[19] P. Samarawickrama , R. Dulal , Z. Fu , U. Erugu , W. Wang , J. Ackerman , B. Leonard , J. Tang , T. Chien , J. Tian , ACS omega 2021, 6, 2966. 33553915 10.1021/acsomega.0c05327 PMC7860099

[20] L. Wang , I. Meric , P. Y. Huang , Q. Gao , Y. Gao , H. Tran , T. Taniguchi , K. Watanabe , L. M. Campos , D. A. Muller , J. Guo , P. Kim , J. Hone , K. L. Shepard , C. R. Dean , Science 2013, 342, 614. 24179223 10.1126/science.1244358

[21] X. Cui , G.‐H. Lee , Y. D. Kim , G. Arefe , P. Y. Huang , C.‐H. Lee , D. A. Chenet , X. Zhang , L. Wang , F. Ye , F. Pizzocchero , B. S. Jessen , K. Watanabe , T. Taniguchi , D. A. Muller , T. Low , P. Kim , J. Hone , Nat. Nanotechnol. 2015, 10, 534. 25915194 10.1038/nnano.2015.70

[22] H. Choi , B. H. Moon , J. H. Kim , S. J. Yun , G. H. Han , S.‐g. Lee , H. Z. Gul , Y. H. Lee , ACS nano 2019, 13, 13169. 31714742 10.1021/acsnano.9b05965

[23] Z. Cheng , Y. Yu , S. Singh , K. Price , S. G. Noyce , Y.‐C. Lin , L. Cao , A. D. Franklin , Nano Lett. 2019, 19, 5077. 31283241 10.1021/acs.nanolett.9b01355

[24] C.‐S. Lian , C. Si , W. Duan , Nano Lett. 2020, 21, 709. 33378208 10.1021/acs.nanolett.0c04357

[25] Q. Wu , S. Zhang , H.‐F. Song , M. Troyer , A. A. Soluyanov , Comput. Phys. Commun. 2018, 224, 405.

[26] G. E. Blonder , M. Tinkham , T. M. Klapwijk , Phys. Rev. B 1982, 25, 4515.

[27] R. Soulen Jr , J. Byers , M. Osofsky , B. Nadgorny , T. Ambrose , S. Cheng , P. R. Broussard , C. Tanaka , J. Nowak , J. Moodera , A. Barry , J. M. D. Coey , science 1998, 282, 85. 9756482 10.1126/science.282.5386.85

[28] G. J. Strijkers , Y. Ji , F. Y. Yang , C. L. Chien , J. M. Byers , Phys. Rev. B 2001, 63, 104510. 10.1103/PhysRevLett.86.5585 11415307

[29] Y. Ji , G. J. Strijkers , F. Y. Yang , C. L. Chien , J. M. Byers , A. Anguelouch , G. Xiao , A. Gupta , Phys. Rev. Lett. 2001, 86, 5585. 11415307 10.1103/PhysRevLett.86.5585

[30] Q. Li , C. He , Y. Wang , E. Liu , M. Wang , Y. Wang , J. Zeng , Z. Ma , T. Cao , C. Yi , N. Wang , K. Watanabe , T. Taniguchi , L. Shao , Y. Shi , X. Chen , S. Liang , Q. Wang , F. Miao , Nano Lett. 2018, 18, 7962. 30403355 10.1021/acs.nanolett.8b03924

[31] R. Griessen , W. Hai‐hu , A. J. J. van Dalen , B. Dam , J. Rector , H. G. Schnack , S. Libbrecht , E. Osquiguil , Y. Bruynseraede , Phys. Rev. Lett. 1994, 72, 1910. 10055735 10.1103/PhysRevLett.72.1910

[32] S. R. Ghorbani , X. L. Wang , M. Shahbazi , S. Dou , C. Lin , Appl. Phys. Lett. 2012, 100, 21.

[33] F. Xiang , X. Wang , X. Xun , K. De Silva , Y. Wang , S. Dou , Appl. Phys. Lett. 2013, 102, 15.

[34] S.‐G. Jung , S. Seo , S. Lee , E. D. Bauer , H.‐O. Lee , T. Park , Nat. Commun. 2018, 9, 434. 29382852 10.1038/s41467-018-02899-5 PMC5789853

[35] S. Souma , Y. Machida , T. Sato , T. Takahashi , H. Matsui , S.‐C. Wang , H. Ding , A. Kaminski , J. Campuzano , S. Sasaki , et al., Nature 2003, 423, 65. 12721624 10.1038/nature01619

[36] R. Yu , J.‐X. Zhu , Q. Si , Phys. Rev. B 2014, 89, 024509.

[37] M. Kim , Y. Kozuka , C. Bell , Y. Hikita , H. Y. Hwang , Phys. Rev. B 2012, 86, 085121.

[38] A. Tsen , B. Hunt , Y. Kim , Z. Yuan , S. Jia , R. Cava , J. Hone , P. Kim , C. Dean , A. Pasupathy , Nat. Phys. 2016, 12, 208.

