# Supplementary material for: Electrical Step‐Edge Contact to a Topological Superconductor Candidate 2M‐WS2
Source: Adv Sci (Weinh). 2025 Nov 14;13(2):e08242. doi: 10.1002/advs.202508242 (PMC12786309; doi:10.1002/advs.202508242)
Supplement: Supplementary file 1 — Supporting Information [file ADVS-13-e08242-s001.pdf]

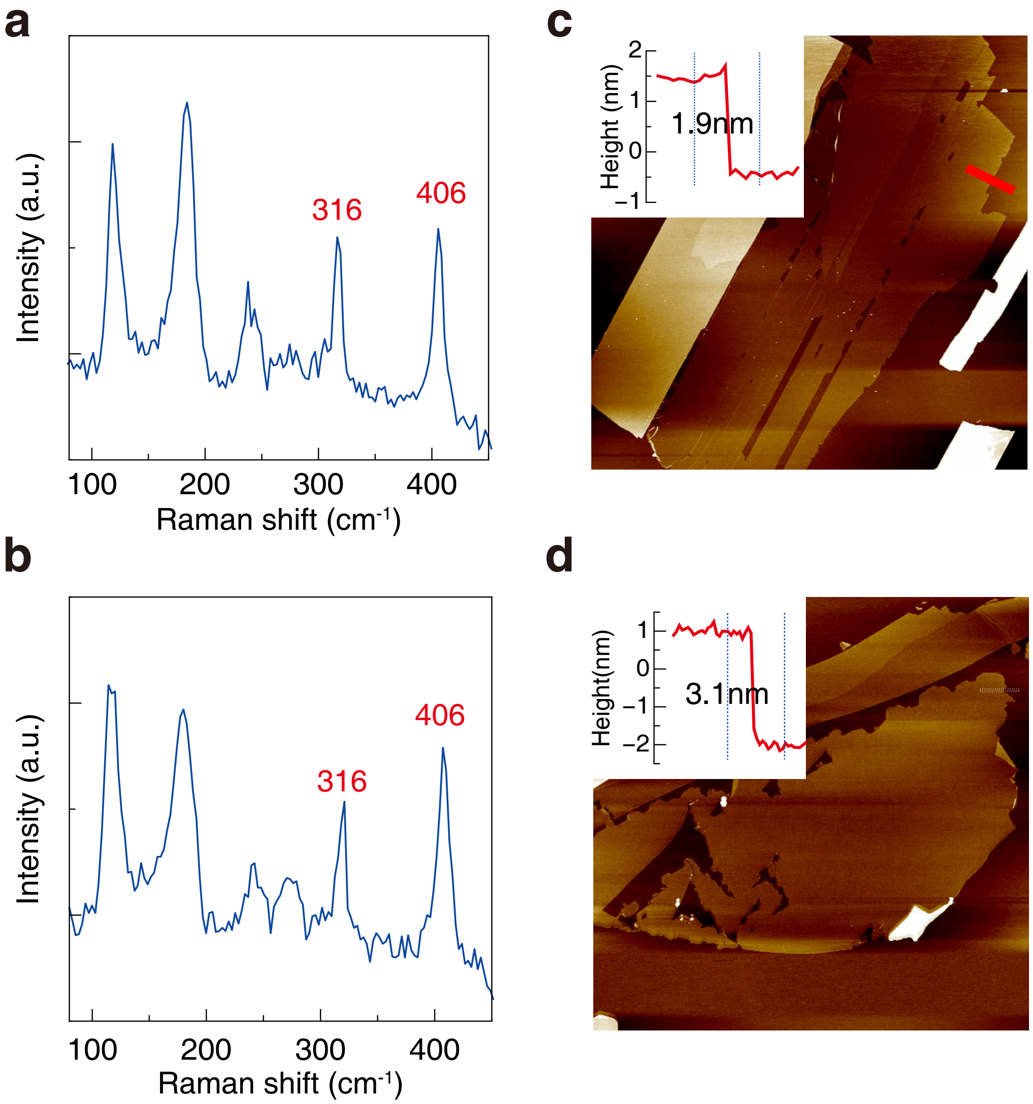

Figure S1. **Raman and Atomic Force Microscope (AFM) measurements of exfoliated 2D 2M-WS$_2$. a-b**, The Raman spectrum of the 1 unit (**a**) and 2 units (**b**) 2M-WS2 crystal. **c-d**, The thickness measurement along the scanning direction, corresponding to the red line in (a-d), respectively. The atomic-level surface flatness demonstrate the high quality of our devices.

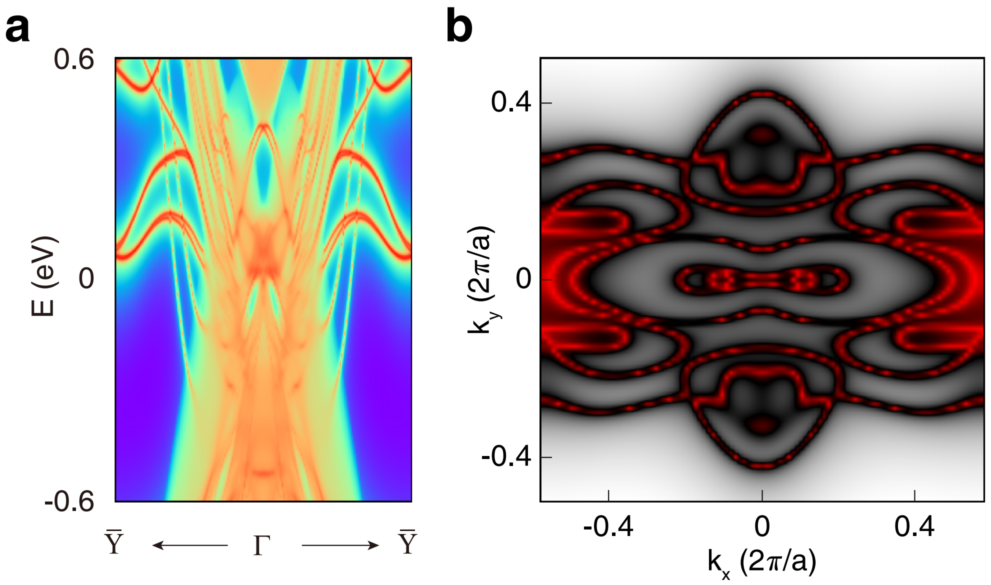

Figure S2. **Calculated energy band of few-layers 2M-WS_2.** **a**, Calculated energy band of three units 2M-WS_2. **b**, Calculated Fermi surface of two units 2M-WS_2, in which multiple Fermi pockets are shown.

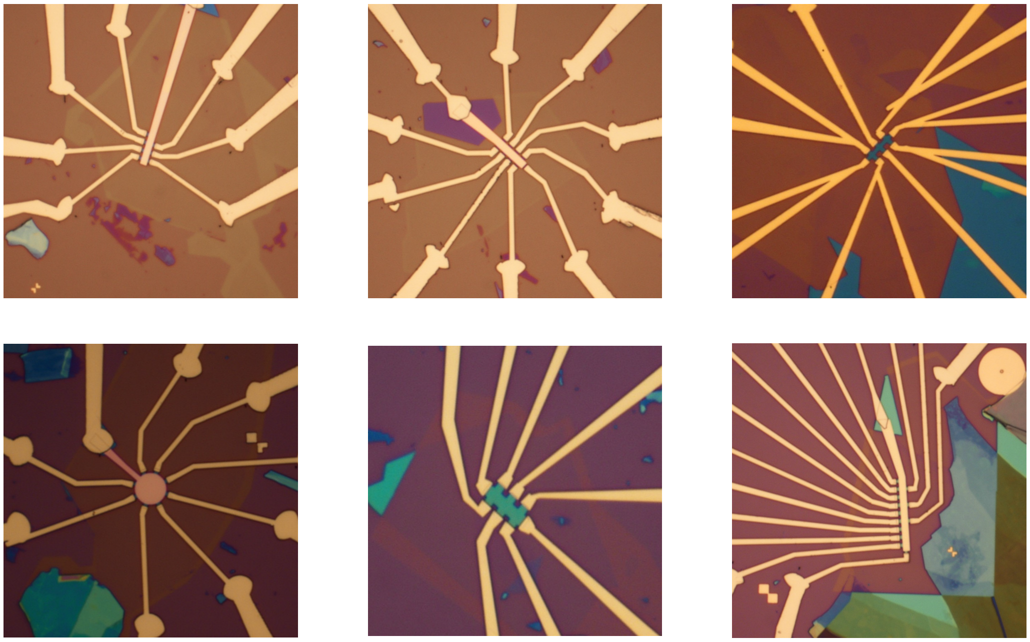

Figure S3. **Optical image of devices with electrical step-edge contacts.**

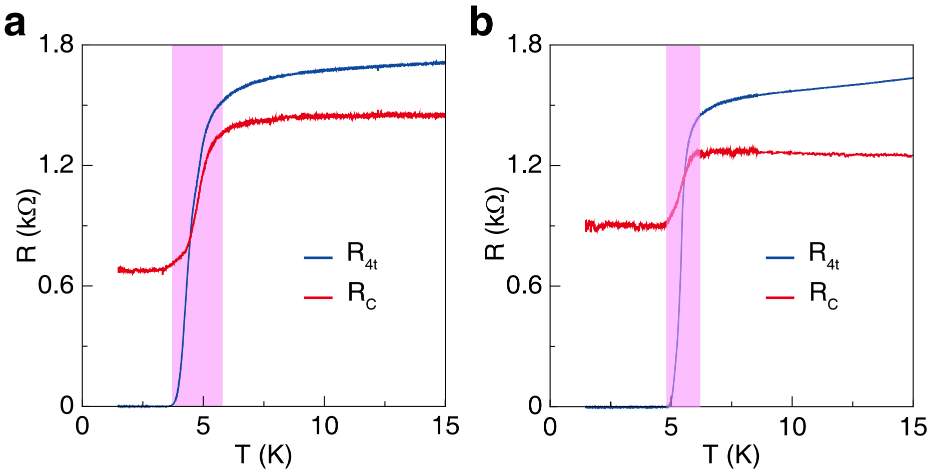

Figure S4. **Step-drop of channel resistance and contact resistance**. **a-b**, Temperature dependence of four terminal and contact resistance of a 1 unit flake device (**a**) and a 2 units flake device (**b**). The drop of contact resistance is consistent with the metal-superconductor transition (pink region).

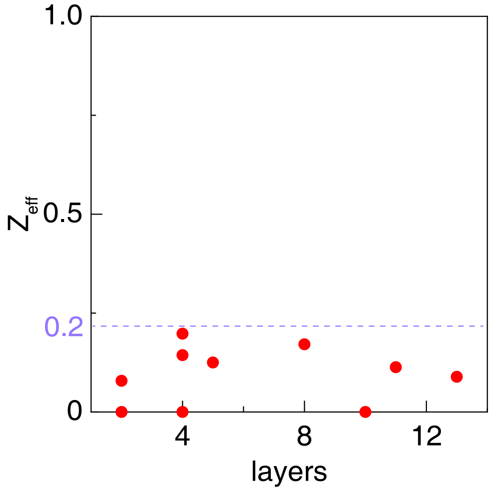

Figure S5. **Calculated effective barrier strength versus layer of 2M-WS_2 among all devices measurements**. The all values are smaller than 0.2 which demonstrates the high efficiency of the edge contact geometry.

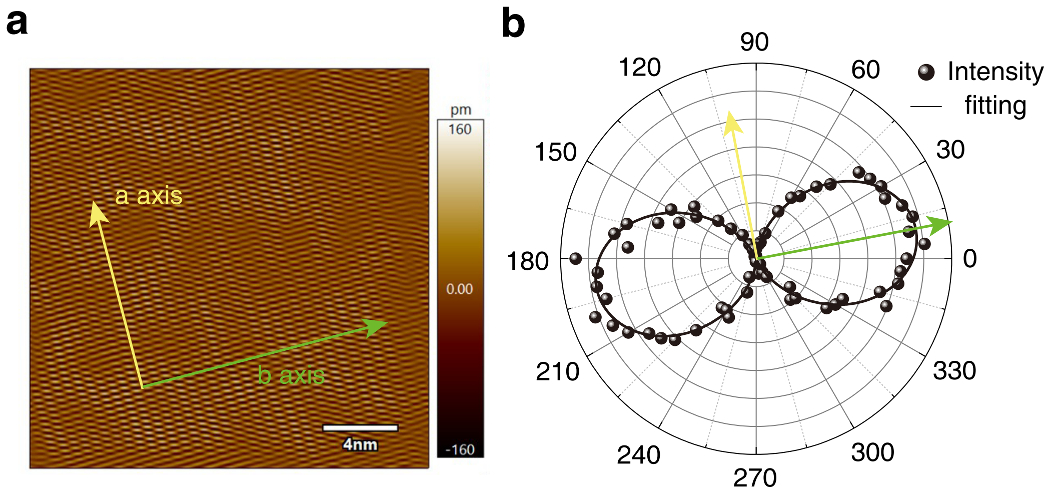

Figure S6. **Comparison of Lateral Force Microscope (LFM) and angle-dependent Raman measurements**. **a**, The hight image of LFM measurement in 2M-WS_2. The arrows represent the orientation of the crystal lattice. **b**, Angular-dependence of Raman mode at 406cm^{-1} measured in the same material as in panel **a**. The Raman intensity is maximized along the *b*-axis.

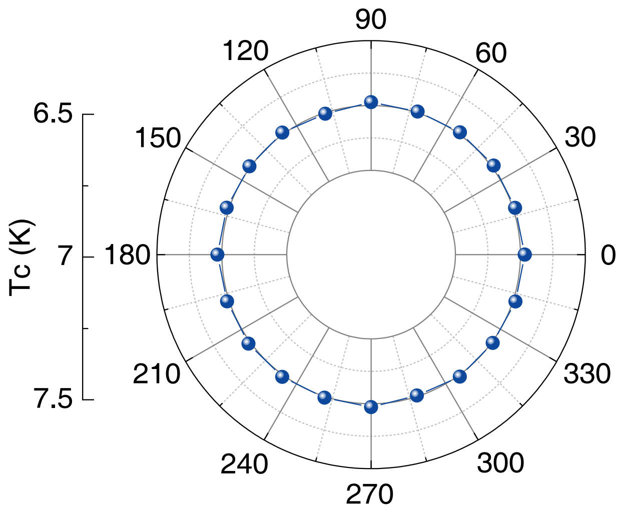

Figure S7. **Angular dependence of critical temperature**. The isotropic behavior at the critical temperature is observed around 7 K.

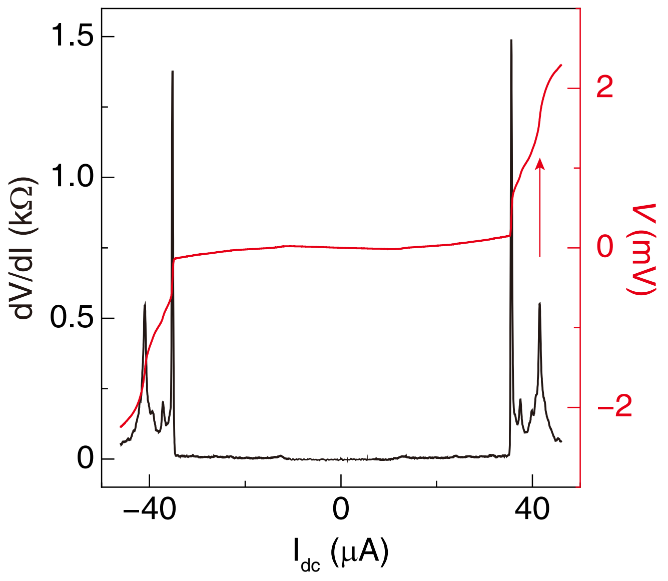

Figure S8. **Measured dV/dI and integrated V as functions of current**. The voltage value corresponding to the outermost peak (red arrow) is around 1.5 mV. It is also noticed that the critical currents for positive and negative directions are not symmetric, implying some possible superconducting diode effect.

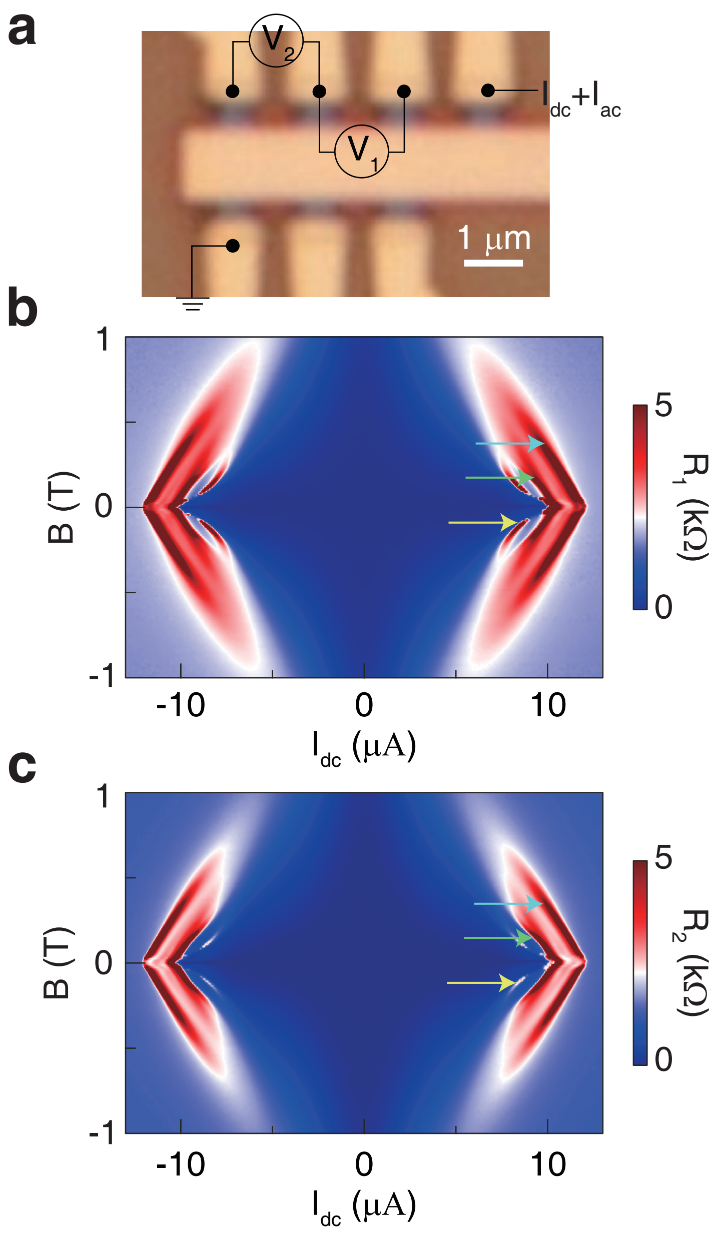

Figure S9. **Multiple peaks in the differential resistance spectra of the multi-channel device**. **a**, Optical micrograph of the device and a schematic of the transport measurement setup. **b-c**, Map of dV/dI versus Idc and B for (b) channel 1 and (c) channel 2.

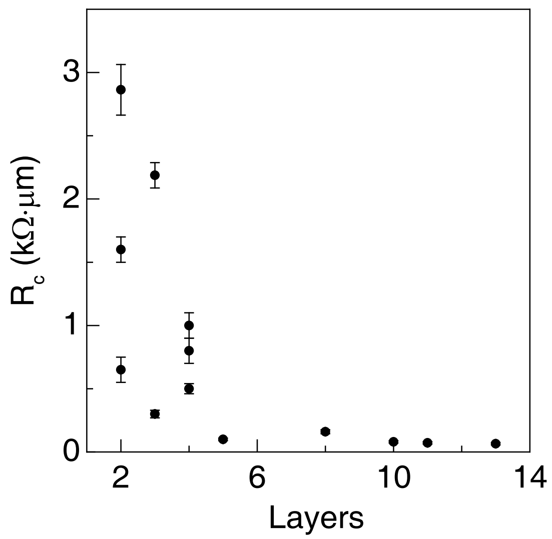

Figure S10. RC of all devices.

	I_{C1}	I_{C2}	I_{C3}	I_{C4}
$I_C(0)$ (μA)	9.64	19.06	26.02	30.94
$T_C (K)$	4.07	5.18	4.71	5.23
α	0.60	0.58	0.56	0.61
β	-0.08	0.38	0.52	0.33

TABLE S1. **Fitting parameters of $I_C - T$ curves.**
